# A dataset of healthcare systems for cross-efficiency evaluation in the presence of flexible measure

**DOI:** 10.1016/j.dib.2019.104239

**Published:** 2019-07-06

**Authors:** Sepideh Abolghasem, Mehdi Toloo, Santiago Amézquita

**Affiliations:** a Department of Industrial Engineering, Universidad de los Andes, Bogotá, Colombia; b Department of Systems Engineering, Faculty of Economics, VŠB-Technical University of Ostrava, Czech Republic

**Keywords:** Healthcare system, Efficiency measure, Data envelopment analysis

## Abstract

This article presents the dataset of the healthcare systems indicators of 120 countries during 2010–2017, which is related to the research article “Cross-efficiency evaluation in the presence of flexible measures with an application to healthcare systems” [1]. The data is collected from the World Bank and selected for the 120 countries. Depending on their role in the performance of the healthcare systems, the indicators are categorized into input (I), output (O) and flexible measure (FM) where the FM measure can play either role of input or output in the healthcare system. The dataset can be used to perform efficiency as well as cross-efficiency analysis of the healthcare systems using methods such as data envelopment analysis (DEA) in the presence of flexible measure.

**Table:** Specifications table

Subject area	Operations research and management science
More specific subject area	Data envelopment analysis
Type of data	Table
How data was acquired	Using a macro developed in Excel Visual Basic to acquire data which is available on World bank open data
Data format	Raw, analyzed with descriptive and statistical data
Experimental factors	The most updated data of healthcare systems for 120 countries available from 2010 to 2017.
Experimental features	indicators of interest were selected and collated.
Data source location	Global data
Data accessibility	Data is within this article and also accessible from the database of the World Bank open data: https://data.worldbank.org
Related research article	S. Abolghasem, M. Toloo, S. Amézquita, “Cross-efficiency evaluation in the presence of flexible measures with an application to healthcare systems,” *Health Care Manag. Sc.*, 2019, 1–22 [1].

**Table:** 

**Value of the data** • The raw data contains the indicators for healthcare systems of 120 countries selected during 2010–2017, which can be used for performance assessment of the countries in terms of their efficiency in their healthcare system in comparison to their peers. • The provided data is useful for decision makers to perform efficiency analysis using methodologies such as data envelopment analysis on the healthcare systems of the 120 countries. • The data is worthwhile to the researchers for efficiency as well as cross-efficiency evaluation of the healthcare systems for the 120 countries under consideration. • The data is useful to evaluate a wide range of efficiency measures for the 120 countries under consideration besides comparative analysis of continental performance and beyond.

## 1 Data

**Fig. 1:**
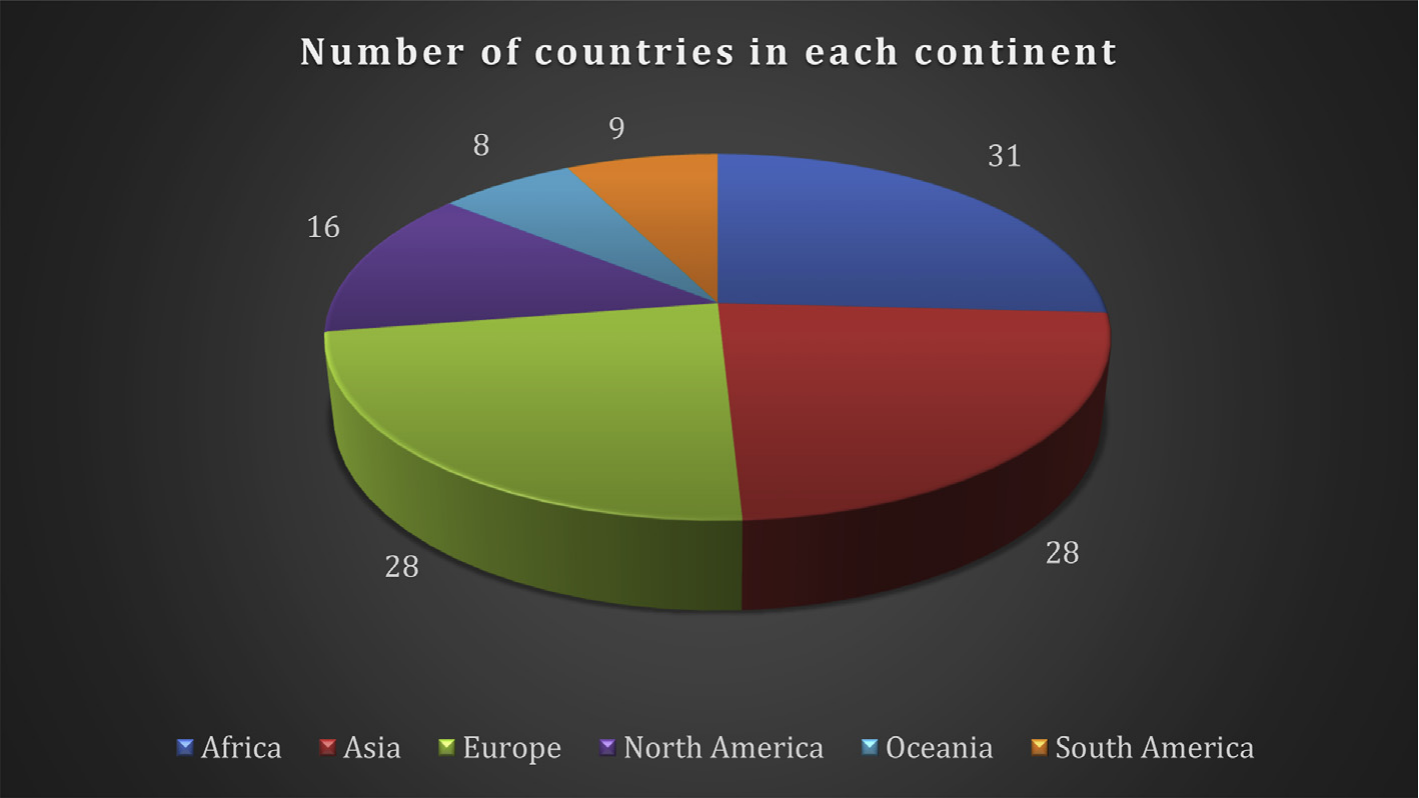
Distribution of countries within the continents.

The data comprises various indicators of the healthcare systems in 120 countries which are selected according to their availability of the data in the World Bank [2] during 2010–2017. The distribution of the selected countries among the continents is shown in Fig. 1. The indicators and their type: input (I), output (O), or flexible measure (FM), as well as the summary of descriptive statistics of the indicators, are provided in Table 1, Table 2, Table 3, Table 4, Table 5, Table 6 for each continent.

**Table 1:** Descriptive statistics of inputs, outputs and flexible measures for Africa.

	Mean	Std. Dev.	Min	Max
***Inputs***				
Population	32237224.5	38877689.9	94677	185989640
Specialist surgical	5.41	11.96	0.17	50.08
Birthrate	33.35	8.93	10.40	48.14
Total Fertility Rate	4.43	1.38	1.40	7.24
Hospital beds	1.39	1.27	0.10	6.30
Nurses and midwives	1.19	1.34	0.08	5.23
Physicians	0.45	0.80	0.02	3.06
***Output***				
Mortality	0.52	0.18	0.32	0.93
***Flexible Measure***				
Life expectancy	62.84	6.54	52.17	75.82

**Table 2:** Descriptive statistics of inputs, outputs and flexible measures for Asia.

	Mean	Std. Dev.	Min	Max
***Inputs***				
Population	86283051.8	247794083.2	427756	1324171354
Specialist surgical	36.21	36.03	0.03	125.01
Birthrate	19.61	6.82	7.80	33.21
Total Fertility Rate	2.41	0.84	1.24	4.64
Hospital beds	4.08	3.61	0.50	13.70
Nurses and midwives	4.15	3.28	0.24	12.50
Physicians	1.84	1.25	0.08	4.78
***Output***				
Mortality	0.83	0.10	0.43	0.97
***Flexible Measure***				
Life expectancy	72.99	5.45	61.16	83.98

**Table 3:** Descriptive statistics of inputs, outputs and flexible measures for Europe.

	Mean	Std. Dev.	Min	Max
***Inputs***				
Population	20949260.07	32028667.21	437418	144342396
Specialist surgical	88.86	31.50	0.81	166.81
Birthrate	10.59	1.28	7.80	32.22
Total Fertility Rate	1.57	0.20	1.24	4.10
Hospital beds	5.44	2.13	0.60	11.30
Nurses and midwives	8.69	3.99	0.70	18.23
Physicians	3.50	0.94	0.31	6.26
***Output***				
Mortality	0.96	0.02	0.75	0.99
***Flexible Measure***				
Life expectancy	78.82	3.84	64.74	82.90

**Table 4:** Descriptive statistics of inputs, outputs and flexible measures for North America.

	Mean	Std. Dev.	Min	Max
***Inputs***				
Population	26023144.56	79772622.31	100963	45004645
Specialist surgical	19.37	12.80	3.40	113.12
Birthrate	17.18	4.61	10.30	25.27
Total Fertility Rate	2.12	0.45	1.40	2.97
Hospital beds	2.28	1.56	0.60	9.00
Nurses and midwives	3.21	2.92	0.10	11.88
Physicians	1.05	0.80	0.10	4.19
***Output***				
Mortality	0.86	0.07	0.69	0.96
***Flexible Measure***				
Life expectancy	74.76	4.38	63.33	82.30

**Table 5:** Descriptive statistics of inputs, outputs and flexible measures for Oceania.

	Mean	Std. Dev.	Min	Max
***Inputs***				
Population	1442360.63	2715989.62	109643	323127513
Specialist surgical	3.70	2.00	2.30	54.71
Birthrate	26.69	4.51	12.40	28.71
Total Fertility Rate	3.76	0.84	1.75	3.85
Hospital beds	2.49	1.68	1.30	5.20
Nurses and midwives	2.35	1.37	0.53	9.88
Physicians	0.32	0.27	0.06	2.57
***Output***				
Mortality	0.77	0.12	0.66	0.95
***Flexible Measure***				
Life expectancy	70.24	3.24	65.54	78.69

**Table 6:** Descriptive statistics of inputs, outputs and flexible measures for South America.

	Mean	Std. Dev.	Min	Max
***Inputs***				
Population	43378930.22	64011970.53	107122	207652865
Specialist surgical	27.97	17.59	0.58	61.12
Birthrate	17.87	2.79	14.16	35.05
Total Fertility Rate	2.23	0.30	1.73	5.50
Hospital beds	2.04	1.10	1.00	5.90
Nurses and midwives	2.68	2.47	1.08	7.44
Physicians	1.95	1.19	0.08	3.91
***Output***				
Mortality	0.86	0.05	0.56	0.90
***Flexible Measure***				
Life expectancy	74.38	3.19	68.88	76.58

## 2 Experimental design, materials and methods

**Fig. 2:**
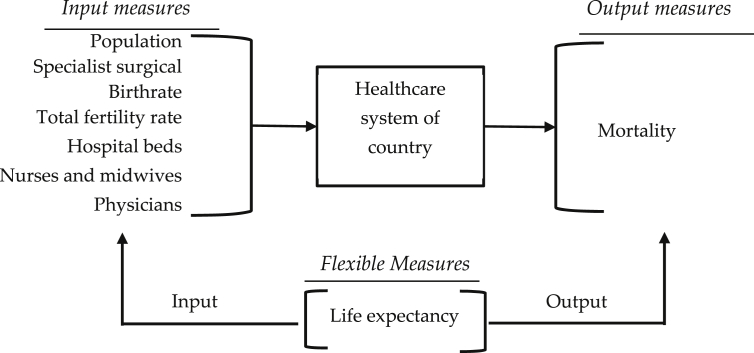
Input, output, and flexible measures with countries as DMUs.

The data for the healthcare systems was collected from the World Bank [2] containing information for different indicators. Then according to the availability of the information during 2010–2017, the dataset was selected and compiled for the 120 countries. The countries are arranged in the ascending order of their Decision-Making Unit identity (DMU ID) in the first column. The DMU ID, starting from 1 to 120, corresponds to the country name organized in alphabetical order. Subsequently, for the performance analysis of the healthcare systems using the data envelopment analysis (DEA) methodology, the indicators are categorized into input (I), output (O), and flexible measure (FM) according to Fig. 2.

The healthcare system measures were divided into three categories based on their role in the performance of the healthcare system. The population, specialist surgical, birthrate, total fertility rate, hospital beds, nurses and midwives, physicians were categorized as the input of the study and mortality was treated as the output. The aforementioned categorization is in accordance with similar studies on the healthcare system performance in literature [3].

The categorization of the indicators was done according to their natural impact on the performance of the healthcare system. For instance, population, birthrate and total fertility rate were categorized as input since it is supposed that lower level of population, birthrate, and fertility rate results in better housing, nutrition, and access to healthcare. Besides, we categorized specialist surgical, hospital beds, nurses and midwives, and physicians as input since it is preferred that the healthcare system achieve the maximum performance requiring the minimum number of specialist surgical workforce, hospital beds, nurses and midwives, and physicians. Mortality was selected as the output of the healthcare system as by definition it is considered to be a direct measure on the performance of the healthcare system and finally, life expectancy was categorized as the only flexible measure of the study.

It is noteworthy that regarding the selected input, we performed a Pearson correlation analysis, which is a measure of the strength of the association among the measures. The results of the correlation analysis revealed no correlation among the inputs. It should be noted that with the aim to prevent scaling problems of the data, we transformed all the data, by dividing each value of the data set by the maximum value of the corresponding indicator.

Lastly, we would like to confirm that the provided data excludes any type of statistical or scaling oriented modification of the data. The aforementioned modifications such as standardization of the data was performed for the analysis executed in the main manuscript and are not reflected in the data table provided here.
